# Possible Effect of Concomitant Prokinetics and Herbal Medicines against Nausea in Patients Taking Lubiprostone

**DOI:** 10.1155/2017/3762179

**Published:** 2017-12-07

**Authors:** Takatsugu Yamamoto, Shun Osumi, Daisuke Yanagisawa, Hiroshi Yamato, Hitoshi Aoyagi, Akari Isono, Koichiro Abe, Hiroto Kita

**Affiliations:** Department of Internal Medicine, Teikyo University School of Medicine, Tokyo, Japan

## Abstract

**Background and Aim:**

Lubiprostone is a novel laxative that sometimes causes nausea, but preventive strategies remain unconfirmed.

**Methods:**

We retrospectively chose 126 patients prescribed lubiprostone from 2013 to 2016. Medical records were reviewed to clarify whether nausea developed after administration of the drug. Background characteristics, including concomitant medicines, were also reviewed.

**Results:**

The most common adverse symptom was diarrhea (23.8%). Nausea occurred in 16 patients (12.7%). Patients taking either prokinetics or herbal medicines or both were unlikely to develop nausea (*p* = 0.007).

**Conclusions:**

Concomitant prokinetics and/or herbal medicines may help alleviate lubiprostone-induced nausea.

## 1. Introduction

Chronic constipation (CC) is a common condition that sometimes impairs a patient's quality of life. The number of patients with CC has recently increased in association with the advanced-aged society in developed countries [1]. Lubiprostone is a selective type 2 chloride-channel activator that increases the liquid component in the small intestine, enhances bowel motility, and improves constipation. This agent has become essential for clinicians in the management of patients experiencing CC and constipation-associated irritable bowel syndrome [2–4]. On the other hand, lubiprostone has been reported to sometimes cause upper gastrointestinal symptoms such as nausea and vomiting, which is one of chief reasons for discontinuation of the drug [5]. A possible mechanism is that an increase in luminal volume may cause distension of the small intestine, inducing a delay in gastric emptying and thus a sensation of nausea via vagal stimulation [6, 7]. However, it is difficult to predict the development of such troublesome symptoms before administration, and strategies for prevention remain uncertain. We conducted the present study to identify the key clinical factors associated with the development of lubiprostone-induced nausea.

## 2. Materials and Methods

This is a retrospective study using the medical records of patients in Teikyo University Hospital (Tokyo, Japan). First, all 269 patients who had been prescribed lubiprostone (Amitiza®, Mylan EPD, Tokyo) for CC in the Department of Internal Medicine (January 2013 to April 2016) were identified from patient lists. A diagnosis of constipation was made according to the Rome III criteria: bowel movements less than three times per week, hard stools, a sensation of incomplete emptying, or difficulty in evacuation on at least 25% of occasions [8]. Then, 80 patients with an insufficient description of clinical effectiveness and 63 patients without follow-up after administration were excluded, with 126 patients finally enrolled as subjects. Medical records of the subjects were examined by two authors (T. Y. and H. A.), to judge their responsiveness to lubiprostone and development of adverse effects such as nausea, vomiting, diarrhea, or others within 1 month (as the endpoints of the study). If the drug was discontinued, the cause was identified, where possible. In addition, clinical data such as demographics and concomitant medications were examined to evaluate their influence on responsiveness and the development of adverse reactions.

In the statistical analysis, a *p* value < 0.05 was regarded as significant. To clarify significant clinical factors related to responsiveness, univariate and multivariate analyses were performed using logistic regression analysis. All statistical evaluations were made using SPSS Statistics, version 19 (IBM Japan, Tokyo, Japan). This protocol was approved by the Institutional Review Board of Teikyo University prior to the study (TU-16-018).

## 3. Results

Background characteristics of the study subjects are shown in Table 1. The subjects included 61 men and 65 women, and the average age was 65.4 years. It was noted that female patients were significantly younger than male patients. The rate of concomitant laxatives was almost 50%. The response rate reached up to 75.4%, while adverse effects were seen in about 40% of all subjects. Common adverse reactions were diarrhea (30 patients, 23.8%) and nausea, vomiting, or both (16 patients, 12.7%). Others included edema (2 patients), headache (1 patient), and palpitations (1 patient). The rate of discontinuation reached 42% of all the subjects. The major reason for discontinuation was an adverse effect such as nausea or diarrhea.

Table 2 shows the impact of clinical factors on responsiveness and adverse effects, using univariate analysis. Effectiveness was significantly related to older age. Regarding adverse effects, no factors were associated with the development of diarrhea. On the other hand, development of nausea was less frequent in elderly patients and in those treated with prokinetics, such as mosapride, itopride, and/or Japanese herbal medicines, including rikkunshito, daikenchuto, mashiningan, or daiou* (Rhei rhizoma)*, than in those without (Table 2). These two groups of medicines were analyzed together because most of them were concomitantly prescribed. Women tended to be more susceptible to nausea than men during lubiprostone treatment. Multivariate analysis showed that age and concomitant prokinetics or herbal medicines were significant factors related to upper gastrointestinal symptoms.

## 4. Discussion

Gastrointestinal symptoms are cumbersome reactions during treatment with lubiprostone. According to previous data, incidence reached 40%, and the most frequent symptom was nausea [5]. In the present study, we found the incidence was similar, though diarrhea was the most common symptom. Unfortunately, because dose reduction has only a partial prophylactic effect against lubiprostone-induced nausea, discontinuation of the agent is necessary, and preventive treatment has not yet been established [9]. In a recent study from Japan, it was reported that itopride, a prokinetic drug with antidopamine effect, might be useful in alleviating nausea [10]. In the present study, we showed the possible effectiveness of concomitant herbal medicines. Daikenchuto is an herbal medicine that is effective for bloating in patients with CC, probably via action on the gut microbiota [11, 12]. Rikkunshito is a ghrelin enhancer and is effective against upper gastrointestinal symptoms [13, 14]. Daiou, or* Rhei rhizoma*, has been proven to have an inhibitory effect on reflux esophagitis in rats [15]. Although it remains unclear which specific type of medicines was most effective for preventing nausea because of the small number of subjects in the present study, prokinetics and herbal medicines might help prevent nausea induced by lubiprostone. Further prospective studies are required to clarify clinical efficacy.

Limitations must be considered in the interpretation of the present data. One is the retrospective study design. Second, this study was carried out at a single institution, and third, the sample size was small. Further multicenter prospective studies are required to determine which agents are appropriate for preventing lubiprostone-induced nausea.

## Figures and Tables

**Table 1:** Background characteristics and clinical response to lubiprostone.

Number of patients	126
Age (year ± SD)	65.4 ± 15.7
Sex (male/female)	61/65
Mental disorder	13 (10.3%)
Concomitant agents	
Laxatives	69 (54.8%)
Prokinetics/herbal medicines	56 (44.4%)
Probiotics	10 (7.9%)
PPI/H2RA	33 (26.2%)
Effectiveness	95 (75.4%)
Diarrhea	30 (23.8%)
Nausea/vomiting	16 (12.7%)
Discontinuation	53 (42%)

Other adverse effects included edema (2 patients), headache (1 patient), and palpitation (1 patient). SD: standard deviation, PPI: proton pump inhibitor, and H2RA: histamine 2 receptor antagonist.

**Table 2:** Relationship between nausea/vomiting after administration of lubiprostone and clinical factors based on univariate analysis.

Clinical factor	Odds ratio	95% confidence interval	*p* value
Age	0.956	0.924–0.988	0.007
Sex	0.310	0.094–1.021	0.054
Mental disorder	0.759	0.258–6.414	0.759
Laxatives	0.898	0.372–3.082	0.898
Prokinetics/herbal medicines	0.037	0.067–0.920	0.037
Probiotics	0.790	0.088–6.333	0.790
PPI/H2RA	0.364	0.078–1.696	0.198

PPI: proton pump inhibitor and H2RA: histamine 2 receptor antagonist.
